# From Neurophysiological Mechanisms to Rehabilitation After Botulinum Toxin Type A in Post-Stroke Spasticity

**DOI:** 10.3390/toxins18090402

**Published:** 2026-09-20

**Authors:** Bart Eeckhaut, Steven Truijen, Parham Haghshenas, Antriana Roussou, Petros Taflampas, Wim Saeys

**Affiliations:** Department of Rehabilitation Sciences and Physiotherapy, Faculty of Medicine and Health Sciences, University of Antwerp, Universiteitsplein 1, 2610 Antwerpen, Belgium; steven.truijen@uantwerpen.be (S.T.); parham.haghshenas@student.uantwerpen.be (P.H.); antriana.roussou@student.uantwerpen.be (A.R.); petros.taflampas@student.uantwerpen.be (P.T.); wim.saeys@uantwerpen.be (W.S.)

**Keywords:** botulinum toxin, stroke, post-stroke spasticity, neurophysiological effects

## Abstract

Background: Botulinum toxin type A (BoNT-A) is globally recognized as a standard treatment for post-stroke spasticity (PSS), although its secondary mechanisms remain underinvestigated. The objective of this study was to investigate the neurophysiological mechanisms underlying BoNT-A treatment in PSS and explore their implications for mechanism-based rehabilitation. Methods: A systematic review and meta-analysis (PROSPERO registration number ID: CRD420261352230) of 39 studies involving 760 patients was conducted on 18 August 2026, following the Preferred Reporting Items for Systematic Reviews and Meta-Analyses (PRISMA) guidelines. PubMed, Web of Science, Scopus, and Embase were systematically searched using the PICO framework. Where possible, quantitative data were pooled according to predefined neurophysiological constructs, including passive stretch-evoked activity, voluntary activation of the injected muscle, compound muscle action potential (CMAP), reciprocal motor control, normalized Hmax/Mmax, and specific spinal inhibitory mechanisms. Results: Quantitative synthesis demonstrated construct-specific neurophysiological changes following BoNT-A treatment. Passive stretch-evoked activity showed the largest pooled effect (Hedges’ *g* = 0.80, 95% CI 0.35–1.24), followed by CMAP amplitude (*g* = 0.69, 95% CI 0.23–1.14) and reciprocal motor control (*g* = 0.52, 95% CI 0.23–0.81). Effects on voluntary activation of the injected muscle (*g* = 0.31, 95% CI −0.38 to 1.00) and normalized Hmax/Mmax (*g* = 0.22, 95% CI −0.74 to 1.17) were smaller and more heterogeneous. Reciprocal and recurrent inhibition were each represented by single-study estimates, while supraspinal outcomes were synthesized narratively due to methodological heterogeneity. Conclusions: The findings support a predominantly peripheral mechanism of BoNT-A while suggesting possible secondary changes within spinal and supraspinal motor control pathways. However, the certainty of evidence was low to very low. These neurophysiological effects provide a rationale for individualized, mechanism-based rehabilitation during the post-injection therapeutic window, although higher-quality evidence is required to confirm this framework.

## 1. Introduction

Stroke is a sudden neurological event caused by an interruption of cerebral blood flow due to either ischemia or haemorrhage [1]. Following stroke, patients commonly develop sensorimotor deficits, including reduced active range of motion, impaired inter-joint coordination, muscle weakness, and spasticity [2,3]. The latter is generally defined as a velocity-dependent increase in tonic stretch reflexes resulting from reflex hyperexcitability [4]. However, post-stroke muscle overactivity may present within a broader spectrum of positive upper motor neuron syndrome manifestations, including spastic dystonia, pathological co-contraction, and associated reactions [5,6,7]. It may progressively impair motor function, independence, and activities of daily living, highlighting the importance of early identification and management [8]. Botulinum toxin type A (BoNT-A) is the primary pharmacological treatment for focal post-stroke spasticity (PSS). By blocking the presynaptic release of acetylcholine at the neuromuscular junction, it reduces excessive muscle activity [9,10].

BoNT-A treatment efficacy is commonly evaluated using clinical outcome measures such as the Modified Ashworth Scale (MAS) [11]. Although other clinical scales provide valuable information on muscle tone, motor impairment, and functional ability, the MAS primarily quantifies resistance to passive movement and cannot adequately distinguish reflex-mediated neural overactivity from non-neural mechanical contributions [12]. Consequently, these measures provide limited insight into the neurophysiological mechanisms underlying treatment effects. Objective neurophysiological assessments provide a complementary approach by quantifying physiological processes involved in motor output and reflex excitability. Electromyographic (EMG) and Hoffmann-reflex (H-reflex) measures can characterize muscle activation and spinal reflex excitability respectively, thereby providing information on treatment-related neurophysiological long-term changes that cannot be derived from clinical scales alone [13,14,15,16].

This distinction is particularly relevant because, although the clinical effectiveness of BoNT-A in reducing PSS is well established, its neurophysiological effects may extend beyond the expected local neuromuscular blockade [10,17,18,19,20]. By reducing muscle activity and modifying afferent input from the injected muscle, BoNT-A may secondarily influence spinal sensorimotor processing and cortical organization [20]. Nevertheless, compared with the extensive literature evaluating clinical outcomes such as muscle tone, passive resistance, range of motion, and function, these spinal and supraspinal mechanisms are substantially less investigated, as the available evidence remains heterogeneous [10,17,18,19,20]. Consequently, it remains unclear which neurophysiological changes effectively occur following BoNT-A in PSS, at which levels of the motor system they occur, how effects across these levels are related, and whether they contribute to short- or longer-term motor outcomes. Addressing this mechanistic gap is clinically relevant because characterizing neurophysiological changes associated with BoNT-A treatment may help explain variability in treatment response, improve the selection of mechanistically relevant outcome measures, and provide a rationale for investigating mechanism-based multidisciplinary rehabilitation during the post-injection therapeutic period. Within this framework, peripheral mechanisms refer primarily to changes at the neuromuscular level, spinal mechanisms to changes in reflex excitability and spinal inhibitory control, and supraspinal mechanisms to changes in cortical activity, connectivity, and sensorimotor organization.

Using the PICO framework, the a priori research question was whether, in adults with post-stroke spasticity (Population), BoNT-A treatment (Intervention), compared with pre-treatment status and/or a comparator condition when available (Comparator), is associated with changes in objective neurophysiological outcomes at the peripheral, spinal, and supraspinal levels (Outcome). This systematic review addresses this gap by integrating the available evidence on BoNT-A-associated neurophysiological changes across peripheral, spinal, and supraspinal levels within a single mechanistic framework. Therefore, the primary aim of this systematic review and meta-analysis was to synthesize the available evidence on neurophysiological changes following BoNT-A treatment in individuals with PSS across peripheral, spinal, and supraspinal levels. Where physiologically and methodologically comparable outcomes were available, treatment effects were quantified by means of meta-analysis, while heterogeneous evidence was synthesized narratively. A secondary aim was to examine how these neurophysiological findings may inform mechanism-based multidisciplinary rehabilitation following BoNT-A treatment.

## 2. Results

### 2.1. Study Selection

A total of 2301 records were identified across four databases. Following consensus, 38 studies identified through database searching met the inclusion criteria. Backward citation searching identified one additional eligible study, resulting in 39 included studies.

### 2.2. Study Characteristics

A total of 39 studies met the inclusion criteria, comprising 760 participants across the included reports. The studies comprised randomized controlled trials, prospective cohort studies, pre–post intervention studies, and observational neurophysiological investigations, reflecting the exploratory nature of the available evidence. Participant age varied considerably across studies, with most study populations consisting of middle-aged to older adults. Time since stroke ranged from the early subacute phase to more than 10 years post-stroke, although the majority of studies investigated chronic post-stroke populations. BoNT-A treatment protocols, injected muscles, neurophysiological outcome measures, follow-up time points, and concomitant rehabilitation interventions varied considerably across studies. Detailed study characteristics are presented in Table 1.

### 2.3. Risk of Bias Assessment

Among the seven randomized trials, risk-of-bias judgements varied across studies, with concerns primarily related to insufficient reporting of randomization and allocation procedures, deviations from the intended interventions, lack of blinding, missing outcome data, and potential bias in outcome measurement. Because blinding of participants and treating clinicians is often difficult in BoNT-A rehabilitation studies, particular attention was given to assessor blinding and the objectivity of the neurophysiological measurements.

Non-randomized studies were assessed according to study design. Comparative studies were evaluated using the Newcastle–Ottawa Scale (NOS), with the main limitations related to cohort selection and comparability between groups. The 26 uncontrolled pre–post studies were assessed using the NHLBI Quality Assessment Tool for Before–After (Pre–Post) Studies With No Control Group (Table 2). Most were judged to be of fair methodological quality, with one study rated as poor. Recurring limitations included small or insufficiently justified sample sizes, incomplete reporting of participant selection and assessor blinding, and the absence of repeated pre-intervention measurements. Moreover, the uncontrolled design limited adjustment for temporal changes and potential confounding by stroke chronicity, baseline motor impairment, BoNT-A dose, and concomitant rehabilitation, thereby limiting causal attribution of the observed neurophysiological changes specifically to BoNT-A.

### 2.4. Certainty Assessment

The certainty of evidence was evaluated using the GRADE domains of risk of bias, inconsistency, indirectness, imprecision, and publication bias (Table 3). For the principal neurophysiological outcomes, including passive and voluntary EMG, H-reflex measures, Hmax/Mmax ratios, reciprocal motor-control outcomes, and related electrophysiological measures, the overall certainty of evidence was rated as very low. This rating reflected serious concerns regarding risk of bias, substantial methodological and statistical inconsistency, and serious imprecision resulting from small sample sizes, wide confidence intervals, and the limited number of studies within each mechanistic pool. Indirectness was not considered a major concern because the included studies generally assessed adults with PSS receiving BoNT-A and measured outcomes relevant to the aim of the review.

### 2.5. Quantitative Synthesis

**Figure 1:**
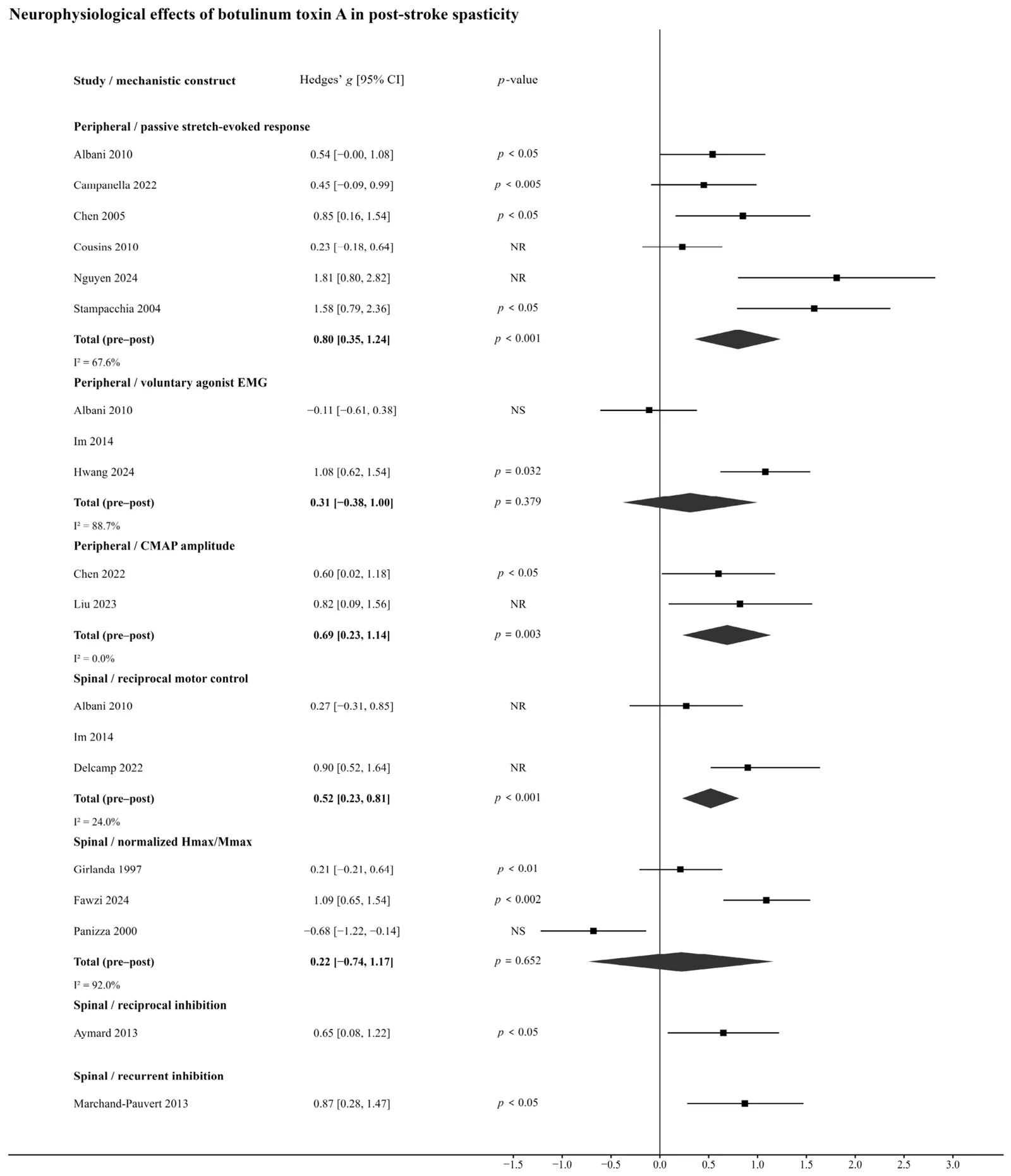
Forest plot of construct-specific neurophysiological effects following BoNT-A in post-stroke spasticity. Individual study effects are expressed as Hedges’ *g* with 95% confidence intervals (CIs). Pooled estimates were calculated using random-effects models and are presented as Total (pre–post) for each mechanistic construct; heterogeneity is reported using the I^2^ statistic. Study-level *p*-values are reported as provided in the original articles; NR indicates not reported and NS indicates not significant. The vertical line at Hedges’ *g* = 0 represents no standardized pre–post change. Reciprocal and recurrent inhibition were represented by single-study estimates and were therefore not pooled. Studies included in the quantitative synthesis were Albani et al. [21], Aymard et al. [23], Campanella et al. [26], Chen et al. [30,31], Cousins et al. [32], Delcamp et al. [33], Fawzi et al. [34], Girlanda et al. [37], Hwang et al. [39], Im et al. [40], Liu et al. [44], Marchand-Pauvert et al. [45], Nguyen et al. [48], Panizza et al. [50], and Stampacchia et al. [51].Abbreviations: BoNT-A, botulinum toxin type A; CI, confidence interval; CMAP, compound muscle action potential; EMG, electromyography; Hmax, maximal H-reflex amplitude; Mmax, maximal M-wave amplitude; NR, not reported; NS, not significant.

Quantitative synthesis was performed for neurophysiological outcomes that were sufficiently comparable with respect to the physiological construct and measurement approach. Outcomes were grouped into passive stretch-evoked responses, voluntary agonist muscle activity, compound muscle action potential (CMAP), reciprocal motor control, normalized H-reflex amplitude (Hmax/Mmax), reciprocal inhibition, and recurrent inhibition. Random-effects models were used where at least two studies could be pooled. Effect sizes are presented as Hedges’ g with 95% confidence intervals (CIs). No overall effect estimate across neurophysiological domains was calculated. An overview of the main quantit ative findings can be seen in Figure 1.

#### 2.5.1. Passive Stretch-Evoked Responses

Six studies contributed to the quantitative synthesis of neurophysiological responses during passive stretch [21,26,30,32,48,51]. The random-effects model yielded a pooled effect of Hedges’ g = 0.80 (95% CI 0.35 to 1.24), with substantial between-study heterogeneity (I^2^ = 67.6%). One study contributed passive stretch-evoked sEMG measurements obtained four weeks after BoNT-A treatment [32].

#### 2.5.2. Voluntary Agonist Muscle Activity

Three studies contributed to the quantitative synthesis of voluntary agonist EMG activity [21,39,40]. Individual effect estimates were g = −0.11 (95% CI −0.61 to 0.38), g = −0.00 (95% CI −0.27 to 0.27), and g = 1.08 (95% CI 0.62 to 1.54), respectively. The pooled effect was Hedges’ g = 0.31 (95% CI −0.38 to 1.00), with substantial between-study heterogeneity (I^2^ = 88.7%). The confidence interval crossed zero and between-study heterogeneity was considerable (I^2^ = 88.7%), indicating substantial uncertainty regarding both the magnitude and direction of the pooled effect.

#### 2.5.3. Compound Muscle Action Potential

Two studies provided quantitative data for CMAP amplitude [31,44]. The individual effect estimates were g = 0.60 (95% CI 0.02 to 1.18) and g = 0.82 (95% CI 0.09 to 1.56), respectively. The pooled effect was Hedges’ g = 0.69 (95% CI 0.23 to 1.14), with no observed statistical heterogeneity (I^2^ = 0.0%). Given that this pooled estimate was based on only two studies, the finding should be considered exploratory and interpreted cautiously.

#### 2.5.4. Reciprocal Motor Control

Three studies contributed to the quantitative synthesis of reciprocal motor control [21,33,40]. The random-effects model yielded a pooled effect of Hedges’ g = 0.52 (95% CI 0.23 to 0.81), with low between-study heterogeneity (I^2^ = 24.0%). 

#### 2.5.5. Normalized H-Reflex Amplitude (Hmax/Mmax)

Three studies contributed to the quantitative synthesis of normalized H-reflex amplitude [34,37,50]. The pooled effect was Hedges’ g = 0.22 (95% CI −0.74 to 1.17), with substantial between-study heterogeneity (I^2^ = 92.0%). The wide confidence interval crossed zero, and the high heterogeneity indicates substantial uncertainty regarding the magnitude and direction of the pooled effect.

#### 2.5.6. Reciprocal and Recurrent Inhibition

Reciprocal inhibition was quantitatively assessed in Aymard et al. (2013), with an effect estimate of Hedges’ g = 0.65 (95% CI 0.08 to 1.22) [23]. Recurrent inhibition was assessed in Marchand-Pauvert et al. (2013), with an effect estimate of Hedges’ g = 0.87 (95% CI 0.28 to 1.47) [45]. As only one study contributed to each mechanism, pooled estimates were not calculated.

#### 2.5.7. Sensitivity Analysis

Sensitivity analyses using alternative assumed pre–post correlations of r = 0.30 and r = 0.70 resulted in only minor changes in the pooled estimates compared with the primary r = 0.50 analyses, without altering the interpretation of the passive stretch-evoked, voluntary agonist activation, CMAP amplitude, or reciprocal motor-control findings (Table 4).

### 2.6. Narrative Synthesis of Non-Pooled Neurophysiological Outcomes

Several included studies could not be quantitatively synthesized because they assessed neurophysiological outcomes that differed from the predefined pooled constructs or were reported in non-comparable formats. These studies were therefore retained for narrative synthesis. Non-pooled findings included passive resistance components, involving passive resistance components, clonus, passive stretch-related measures, motor coordination, spinal inhibitory mechanisms, and supraspinal outcomes.

#### 2.6.1. Passive Stretch and Peripheral Neurophysiological Outcomes

A reduction for EMG activity during passive movement was reported for spastic elbow flexors following BoNT-A (*p* < 0.05), while another report found reduced gastrocnemius sEMG during slow passive dorsiflexion (*p* < 0.05) [49,59]. Another innovative study found a reduction in the neural component of passive wrist resistance at four weeks, with limited changes in the elastic and viscous components [58]. Trompetto et al. (2008) reported a reduction in the tonic vibration reflex relative to Mmax [53]. At motor-unit level, one study reported a 47 ± 9% increase in median motor-unit action-potential amplitude in five of seven participants and an approximately 20 ± 2% reduction in motor-unit territory area at 2–4 weeks [29].

#### 2.6.2. Voluntary Activation and Motor Coordination

One study found no change in tibialis anterior EMG during active dorsiflexion (g = −0.15, 95% CI −0.46 to 0.17), while another reported an increase in an EMG-derived selective motor-control measure (g = 0.61, 95% CI 0.08–1.14) [40,52]. A functional investigation reported reduced rectus femoris–biceps femoris co-contraction and shorter rectus femoris activation duration during gait [35]. Another study using hand-to-mouth task evaluation, reported changes in upper-limb muscle recruitment [36]. In the quantitative analyses, voluntary agonist activation showed a pooled effect of g = 0.31 (95% CI −0.38 to 1.00), while reciprocal motor control showed g = 0.52 (95% CI 0.23–0.81).

#### 2.6.3. Mechanisms on Spinal Level

Aymard et al. (2013) reported an effect on reciprocal inhibition of g = 0.65 (95% CI 0.08–1.22) [23]. Marchand-Pauvert et al. (2013) reported an effect on recurrent inhibition of g = 0.87 (95% CI 0.28–1.47) [45]. While one study did not find any significant changes in first- or second-phase reciprocal inhibition, another reported increased soleus H-reflex post-activation depression in participants with residual motor control and a further decrease in participants without residual motor control [37,41]. Contrary to the latter, another study reported reductions in H-reflex and M-wave amplitudes without a significant change in Hmax/Mmax [47]. The pooled Hmax/Mmax analysis showed g = 0.22 (95% CI −0.74 to 1.17; I^2^ = 92.0%).

#### 2.6.4. Supraspinal and Cortical Outcomes

Delcamp et al. (2022) reported a reduction in antagonist corticomuscular coherence from 0.52 to 0.27 during the BoNT-A efficacy period [33]. Chalard et al. (2021) reported longitudinal changes in movement-related EEG beta desynchronisation following treatment [27]. Concurrently, Veverka et al. (2016) reported changes in task-related fMRI activation involving bilateral cerebellar, contralesional, occipital, and sensorimotor regions [54]. Later, the same research team found no significant longitudinal change in cortical SEP components while increased functional connectivity between the ipsilesional intraparietal region and contralesional superior parietal cortex during peak treatment efficacy was found [55,56]. Vinehout et al. (2021) reported increased activation at six weeks in contralesional premotor, cingulate, thalamic and cerebellar regions and ipsilesional sensory-integration areas [57]. These supraspinal outcomes were not quantitatively pooled.

## 3. Discussion

The present systematic review aimed to synthesize the neurophysiological effects of BoNT-A in PSS across peripheral, spinal, and supraspinal levels. Overall, the findings demonstrate measurable changes in several neurophysiological constructs following BoNT-A treatment, with the most consistent evidence observed at the peripheral level, while spinal and supraspinal findings were more heterogeneous. The following discussion considers these findings according to their proposed mechanistic level, their relationship with clinical outcomes, and the methodological limitations of the current evidence.

### 3.1. From Neurophysiological Mechanisms to Mechanism-Based Rehabilitation Following BoNT-A

The quantitative synthesis showed that BoNT-A produces measurable but construct-dependent neurophysiological changes. The largest pooled effect was observed for passive stretch-evoked activity across six studies (Hedges’ g = 0.80), followed by inappropriate activation of the injected muscle during antagonist contraction across three studies (g = 0.52). Although the pooled effect for passive stretch-evoked responses was large (Hedges’ *g* = 0.80), this estimate aggregated heterogeneous measurement approaches, including sEMG-derived responses and biomechanical measures of reflex-mediated torque or resistance. Accordingly, the magnitude of this effect should be interpreted cautiously, particularly given the very low certainty of the underlying evidence. Effects on voluntary agonist activation (g = 0.31) and normalized Hmax/Mmax (g = 0.22) were smaller and more variable, while reciprocal inhibition, recurrent inhibition, and supraspinal changes were supported by fewer and methodologically heterogeneous studies. These differences highlight that reducing clinically observed muscle tone does not necessarily represent a uniform change across the underlying neurophysiological mechanisms. Consequently, the relevance of BoNT-A treatment should be considered in relation to the specific impairment targeted and the patient’s functional treatment goals.

Treatment should therefore begin with patient-centered goals rather than the presence of increased muscle tone alone. Current international recommendations emphasize that BoNT-A should be integrated within a multidisciplinary rehabilitation program in which treatment goals are established according to the patient’s needs and may include pain, hygiene, positioning, orthotic tolerance, mobility, task performance or participation [60,61,62]. Following goal setting, assessment should determine whether the principal limitation results from structural–mechanical muscle properties or neural overactivity. As demonstrated in a previous systematic review, these represent distinct contributors to PSS and should therefore be considered separately during clinical decision-making [63]. Likewise, clinical scales such as the Modified Ashworth Scale should be complemented by assessments capable of differentiating neural from mechanical resistance, including the Modified Tardieu Scale and, where available, instrumented stretch-reflex assessment [64,65,66].

The present findings could support a mechanism-based approach to post-injection rehabilitation. However, the proposed rehabilitation strategies are hypothesis-generating implications derived from the observed neurophysiological changes and were not directly evaluated in the included studies. When passive overactivity limits joint mobility, hygiene or orthotic positioning, rehabilitation should focus on improving access to range through stretching, casting, splinting or orthotic management [67]. Conversely, when movement is restricted by inappropriate muscle activation, treatment should selectively reduce the interfering overactivity while preserving useful voluntary recruitment, followed by task-specific rehabilitation aimed at improving movement quality and active function [61]. Most importantly, the observed reduction in inappropriate activation of the injected muscle during antagonist contraction suggests that rehabilitation should specifically target the previously inhibited antagonist through selective activation, progressive strengthening and task-specific motor training. Similarly, patients presenting with pathological co-contraction should receive interventions aimed at restoring reciprocal motor control rather than solely reducing muscle tone. Recent evidence further supports the use of surface EMG co-contraction indices as objective measures of abnormal muscle coordination during stroke rehabilitation [58].

BoNT-A should therefore be regarded as creating a therapeutic window rather than representing the endpoint of treatment. Clinical effects generally develop within the first one to two weeks after injection and persist for approximately three to four months, providing a period during which rehabilitation can exploit the temporary reduction in pathological muscle activity [68,69]. During this period, rehabilitation should be guided by repeated clinical assessment and adapted to the patient’s evolving motor capacity rather than delivered as a predetermined, uniform programme [70]. Limited evidence suggests possible alterations in reciprocal inhibition, recurrent inhibition and sensorimotor connectivity after BoNT-A treatment. However, whether such changes contribute to improved motor learning or rehabilitation responsiveness remains unclear [71,72].

Overall, the findings support a shift from an injection-centered approach toward a goal- and mechanism-based rehabilitation rationale. Rather than applying a uniform rehabilitation programme following BoNT-A, clinicians should identify the predominant mechanism limiting function and select rehabilitation strategies that specifically exploit the temporary reduction in pathological muscle overactivity. Although the available evidence was characterized by small samples, heterogeneous study designs, variable BoNT-A protocols, different injected muscles, follow-up periods, rehabilitation programmes, and neurophysiological methods, these issues were reflected in the risk-of-bias and GRADE assessments, with risk of bias, inconsistency, and imprecision reducing certainty. Therefore, prospective validation is required, as this rationale provides a clinically relevant model for integrating BoNT-A within multidisciplinary post-stroke rehabilitation [70,72].

### 3.2. Limitations

First, case reports and case series were excluded to reduce the risk of bias. However, this could have excluded informative proof-of-concept research. In addition, eligibility decisions were required for overlapping concepts such as spasticity, spastic dystonia, sensory outcomes, and nociceptive responses.

Second, quantitative synthesis required grouping different neurophysiological measures into broader mechanistic constructs. Pooling was restricted to outcomes considered physiologically comparable, but heterogeneity remained in muscles, motor tasks, EMG normalization, stimulation protocols, and assessment timing. Several constructs were therefore based on only two or three studies.

Third, most quantitative estimates were derived from uncontrolled pre–post comparisons, limiting causal attribution of the observed changes specifically to BoNT-A. Time-related changes, concurrent rehabilitation, and other uncontrolled factors cannot be excluded. Incomplete reporting also required conversion or estimation of some numerical data, including assumptions regarding pre–post correlations. Publication bias and small-study effects could not be formally assessed because of the limited number of studies per construct and therefore cannot be excluded.

Finally, the translation of group-level neurophysiological findings into individualized rehabilitation decisions may be subject to ecological bias. Construct-level effects observed across study populations should not be assumed to predict treatment response or identify the optimal rehabilitation strategy for an individual patient; therefore, the proposed translational framework requires prospective validation at the patient level.

### 3.3. Future Research

Future research should move beyond demonstrating reductions in clinical muscle tone and investigate how neurophysiological assessment can be used to individualize post-injection rehabilitation. Prospective studies should classify patients according to the predominant mechanism limiting function, including passive neural overactivity, structural–mechanical muscle changes, impaired voluntary activation, abnormal reciprocal muscle activation, antagonist weakness, and motor coordination deficits.

Larger, adequately powered randomized controlled trials are required with standardized rehabilitation protocols and transparent reporting of treatment timing, intensity, progression, and adherence. Future studies should systematically document and standardize concomitant rehabilitation to better distinguish BoNT-A-related effects from the additional effects of rehabilitation without withholding conventional care.

Comprehensive assessment should integrate neurophysiological and structural–mechanical measures, including EMG, Hmax/Mmax, reciprocal inhibition, TSRT, ultrasound, elastography, and muscle architecture, to identify the mechanisms underlying functional impairment and guide treatment selection.

Longitudinal studies should evaluate patients throughout the entire treatment cycle and determine whether improvements in mobility, antagonist recruitment, motor coordination, and functional performance persist beyond the pharmacological duration of BoNT-A. In addition, the proposed therapeutic window rationale should be prospectively tested by comparing mechanism-based rehabilitation with contemporary post-injection care.

Finally, multimodal longitudinal studies combining neurophysiology, biomechanics such as gait analysis, and neuroimaging are needed to determine whether peripheral reductions in pathological muscle activity facilitate secondary spinal and supraspinal changes and contribute to sustained motor recovery.

## 4. Conclusions

BoNT-A was associated with reduced passive muscle overactivity and less activity of the spastic muscle during antagonist contraction, while effects on voluntary activation and spinal excitability were more variable. These findings support viewing BoNT-A as a means of removing a focal barrier to movement rather than as a restorative treatment itself. Its clinical value may therefore depend more on patient-centered assessment and mechanism-matched multidisciplinary rehabilitation within the hypothesis of a post-injection therapeutic window. Given the low certainty of evidence, this rationale requires prospective validation.

## 5. Materials and Methods

This systematic review and meta-analysis were conducted in accordance with the Preferred Reporting Items for Systematic Reviews and Meta-Analyses (PRISMA 2020) guidelines and prospectively registered in PROSPERO (CRD420261352230) [73]. A final update search has been completed on 18 August 2026, and aimed to evaluate neurophysiological effects of BoNT-A in PSS.

### 5.1. Eligibility Criteria

This meta-analysis included studies that satisfied the following eligibility criteria: (1) participants were adults (≥18 years) diagnosed with stroke as the primary neurological condition, with involvement of upper and/or lower limbs; (2) studies evaluated the effects of botulinum toxin (BoNT-A) injections on neurophysiological outcomes; (3) when available, studies comparing BoNT-A alone vs. combined therapy were included; (4) Studies were eligible if they reported at least one neurophysiological outcome related to PSS following BoNT-A treatment, including electromyographic measures, H-reflex-related outcomes, reflex threshold measures, or other objective neurophysiological assessments. (5) Randomized controlled trials, non-randomized controlled studies, cohort studies, and pre–post intervention studies were eligible except for case reports, case series (N < 5 discarded), systematic reviews, meta-analyses, surveys, and narrative reviews. Because the review aimed to characterize neurophysiological changes associated with BoNT-A treatment rather than exclusively estimate comparative treatment efficacy, studies reporting within-participant pre–post neurophysiological outcomes were eligible even in the absence of a separate control group.

Spasticity was considered within the broader spectrum of positive upper motor neuron syndrome manifestations following stroke and was defined as a velocity-dependent increase in tonic stretch reflexes resulting from reflex hyperexcitability [4]. Other forms of post-stroke involuntary muscle overactivity, including spastic dystonia, co-contraction, and associated reactions, were also considered eligible when assessed using objective neurophysiological measures. Studies investigating cervical dystonia or dystonia unrelated to stroke, as well as facial, bladder, or other non-limb indications, were excluded. Eligible neurophysiological outcomes were objective measures of peripheral, spinal, or supraspinal motor-system function, including EMG, compound muscle action potential/motor response wave (M-wave), motor-unit activity, H-reflex measures, reflex thresholds, spinal inhibitory mechanisms, Transcranial Magnetic Stimulation (TMS), electroencephalogram (EEG), and functional neuroimaging. Outcomes were classified according to the neurophysiological process assessed rather than the measurement technique itself. Purely mechanical or structural muscle outcomes and sensory outcomes primarily assessing pain, nociception, or touch were not considered as neurophysiological outcomes.

### 5.2. Information Sources

A systematic literature search was conducted in PubMed, Web of Science, Scopus, and Embase from database inception on 18 August 2026, using a predefined PICO-based search strategy. The search yielded 2301 records from the four databases. To maximize study identification, backward citation tracking of the reference lists of eligible studies and relevant reviews was performed, only one additional article was found.

### 5.3. Search Strategy

Database-specific searches combined controlled vocabulary, free-text terms, and Boolean operators (Table 5). The final search was conducted on 18 August 2026, with screening and study inclusion definitively closed.

### 5.4. Selection Process

**Figure 2:**
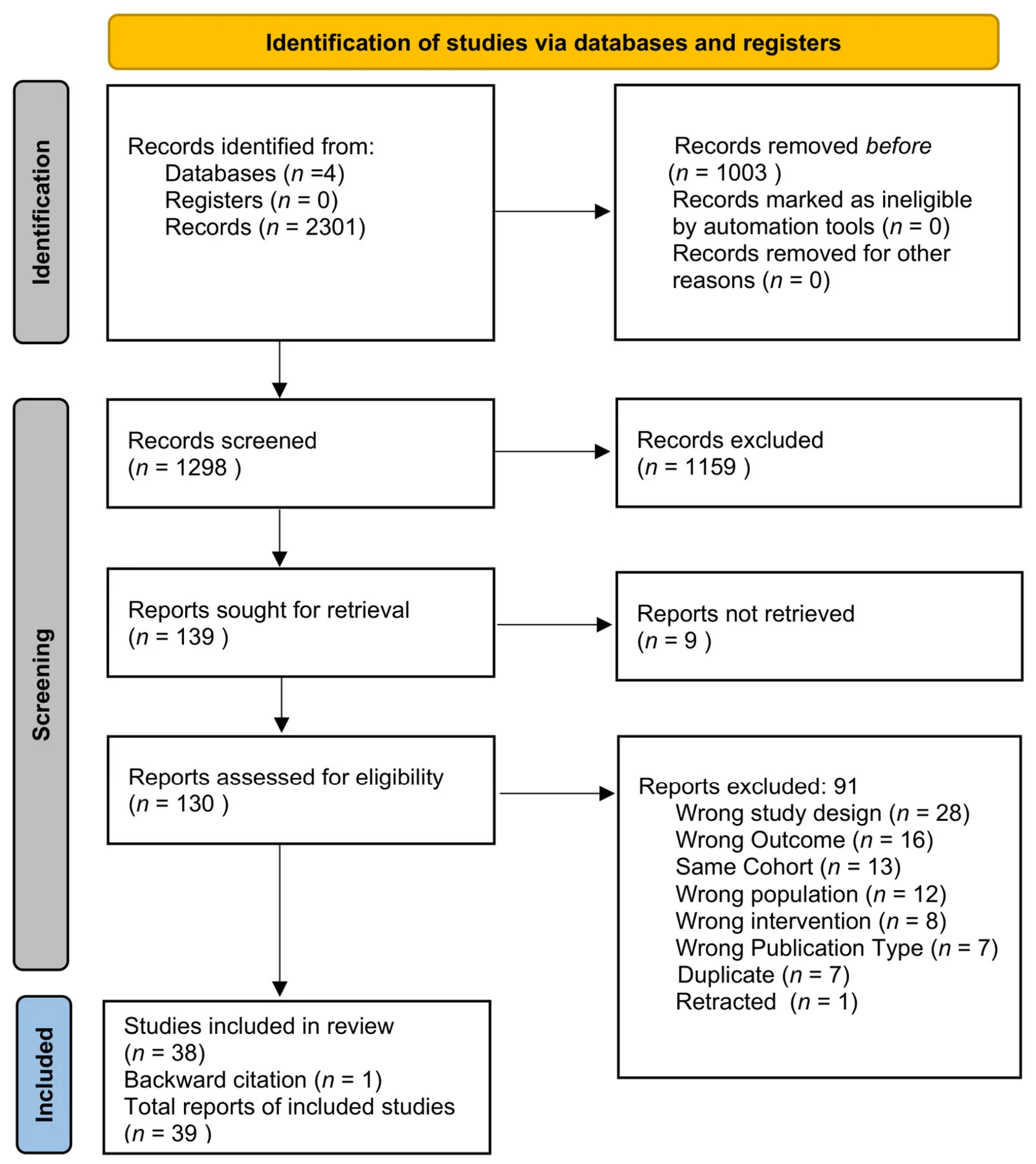
The PRISMA flowchart.

Following keyword and MeSH term selection, all retrieved records were imported into Rayyan (Rayyan Systems Inc., Cambridge, MA, USA) software to remove duplicates across databases and facilitate the two-stage screening process [74]. Three reviewers (P.H., P.T., and A.R.) independently screened titles and abstracts according to the predefined eligibility criteria after it was automatically checked for duplicates by the automated software. Studies deemed potentially eligible proceeded to full-text assessment, which was independently performed by the same reviewers. Disagreements were resolved through discussion and consensus. When consensus could not be reached, a fourth reviewer (WS) was consulted. Disagreements requiring adjudication occurred for 20 full-text articles. Following consensus, 39 studies met the inclusion criteria and were included for data extraction. An overview of the selection process can be found in Figure 2.

### 5.5. Data Collection Process

Data were independently extracted from all included studies using a standardized extraction form. Any uncertainties during data extraction were resolved through discussion among the reviewers (AR, PT, PH & BE). Because the objective of this review was to characterize the neurophysiological mechanisms underlying BoNT-A treatment, outcomes were classified a priori according to the neurophysiological construct they represented rather than solely according to the measurement modality. Only outcomes reflecting comparable neurophysiological mechanisms were pooled within the same meta-analysis.

### 5.6. Data Items

Extracted variables included study characteristics (author, year, study design), participant characteristics (sample size, age, sex, stroke type, stroke chronicity, and affected limb), intervention characteristics (BoNT-A formulation, injected muscles, toxin dose, injection guidance method, and concomitant rehabilitation interventions), comparator interventions where applicable, follow-up time points, and all reported neurophysiological and clinical outcome measures.

Neurophysiological outcomes included measures of voluntary muscle activation, co-contraction, gait-related muscle activation, spinal excitability (H-reflex parameters), corticomuscular coherence, and other peripheral, spinal, or supraspinal motor function assessments. Clinical outcome measures were extracted to contextualize neurophysiological findings but were not included in the primary quantitative synthesis. Any uncertainties or discrepancies during data extraction were resolved through discussion and consensus among the reviewers.

### 5.7. Planned Methods of Analysis

Quantitative synthesis was performed using conventional pairwise meta-analysis methods in accordance with the Cochrane Handbook for Systematic Reviews of Interventions [72]. Continuous neurophysiological outcomes were summarized using standardized effect sizes calculated as Hedges’ g. Meta-analyses were performed using R version 4.4.3. (R Foundation for Statistical Computing, Vienna, Austria) Random-effects models were fitted using the DerSimonian–Laird estimator for between-study variance (τ^2^), and heterogeneity was quantified using the I^2^ statistic. For the construct-specific meta-analyses, neurophysiological effects were expressed as standardized pre–post changes within the BoNT-A-treated group. This approach was selected because most eligible studies used uncontrolled before–after designs and because comparator groups, where present, were heterogeneous and were not consistently designed to estimate the neurophysiological effect of BoNT-A. Standardized mean changes were calculated from pre–post data and corrected for small-sample bias to obtain Hedges’ g. The pre–post correlation reported by the original study was used where available; When the within-participant pre–post correlation was not reported, a correlation of r = 0.50 was assumed for variance estimation. Sensitivity analyses were performed using alternative correlation coefficients of r = 0.30 and r = 0.70 to assess the robustness of the pooled estimates to this assumption. Sensitivity analyses were performed to examine the influence of this assumption on the pooled estimates. Consequently, for controlled studies, only the within-group pre–post change in the BoNT-A-treated group was included in the construct-specific meta-analyses, allowing neurophysiological-related changes to be synthesized consistently across study designs.

Random-effects models were applied because substantial methodological and clinical heterogeneity was anticipated, including differences in participant characteristics, Time Post-Stroke, injected muscles, BoNT-A dosage, rehabilitation co-interventions, neurophysiological assessment protocols, and follow-up duration [75,76].

Because the objective of this review was to investigate neurophysiological mechanisms rather than individual assessment techniques, outcomes were classified a priori according to the physiological construct they represented. For EMG-based outcomes, classification additionally considered the muscle from which activity was recorded and the motor condition under which it was elicited. Activity recorded from the injected/spastic muscle during passive stretch, voluntary contraction of that muscle, and voluntary contraction of its antagonist was therefore treated as three distinct neurophysiological constructs. Outcomes were pooled only when they represented the same physiological construct and were considered sufficiently comparable in terms of muscle function and motor condition. Outcomes that could not be meaningfully combined were synthesized narratively. Quantitative synthesis was performed only when studies assessed sufficiently comparable constructs and provided adequate numerical data for effect-size calculation. Separate analyses were therefore conducted for passive stretch-evoked activity, voluntary agonist activation, compound muscle action potential, reciprocal motor control, normalized Hmax/Mmax, and specific spinal inhibitory mechanisms. When outcomes were represented by too few studies, differed substantially in methodology or physiological construct, or could not be converted into comparable quantitative estimates, they were synthesized narratively. This approach prevented pooling of outcomes that used the same measurement modality, such as EMG, but represented different underlying neurophysiological processes.

Statistical heterogeneity was quantified using the I^2^ statistic, with values of 25%, 50%, and 75% representing low, moderate, and high heterogeneity respectively [77]. Planned sensitivity analyses explored the influence of study design, methodological quality, and assumptions regarding within-subject correlation for standardized mean change calculations on pooled effect estimates. When numerical data were incompletely reported, values were extracted from published figures or reconstructed from available summary statistics whenever possible. Studies without sufficient quantitative information for effect-size calculation were retained in the systematic review and synthesized narratively when they contributed to the predefined mechanistic framework.

Small-study effects and publication bias were not formally assessed using funnel plots or statistical tests such as Egger’s test because fewer than 10 studies contributed to each pooled construct, limiting the reliability and interpretability of these methods.

### 5.8. Risk of Bias Assessment

Risk of bias was independently assessed by three reviewers using the Cochrane Risk of Bias 2 (RoB 2) tool for randomized trials (Table 6), the Newcastle–Ottawa Scale (NOS) for non-randomized comparative studies (Table 7), and the National Heart, Lung, and Blood Institute (NHLBI) Quality Assessment Tool for Before–After (Pre–Post) Studies With No Control Group for uncontrolled pre–post studies [78,79]. 

### 5.9. Certainty Evidence

The certainty of evidence for each outcome domain was assessed using the Grading of Recommendations Assessment, Development, and Evaluation (GRADE) approach [80]. Evidence was evaluated across five domains: risk of bias, inconsistency, indirectness, imprecision, and publication bias. The certainty of evidence was classified as high, moderate, low, or very low, according to GRADE recommendations. GRADE assessments were performed independently by the reviewers, with disagreements resolved through discussion and consensus.

## Figures and Tables

**Table 1:** Evidence Table: Neurophysiological Effects of Botulinum Toxin A in Post-Stroke Spasticity. BoNT-A doses are reported as described in the original studies; potency units are formulation-specific and are not directly interchangeable between botulinum toxin preparations.

Study	Design	Participants	Time Post Stroke	BoNT-A Intervention	Neurophysiological Outcomes	Neurophysiological Outcome Domain	Clinical Outcomes	Results (Uniform, Compact)
Albani et al. (2010) [21]	Pre–post	*n* = 10	1–6 years (chronic)	BB 100U; FCR 50U; FDP 50U	EMG	Peripheral	MAS; GPS	MAS ↓ at 30d, return toward baseline at 180d; GPS ↓ at 30d & 180d (*p* < 0.05); EMG ↓ ~40–50% at rest/passive movement.
Alvisi et al. (2018) [22]	Pre–post	*n* = 14	Subacute/established PSS; exact time NR here	450–1680U (AD, PD, BB, TB, FCR, ECR)	EMG; NWR	Peripheral	MAS; FIM	MAS ↓ (*p* < 0.001); FIM ↑ (*p* < 0.001); EMG ↓ in AD/PD/BB/TB/FCR (*p* < 0.01); NWR ↓ (*p* = 0.015).
Aymard et al. (2013) [23]	Pre–post	*n* = 13	Chronic PSS (≥6 months)	Soleus/MG/TP (300U)	EMG; H-reflex/reciprocal inhibition	Peripheral; spinal	MAS	MAS ↓ ~2 points; EMG ↓ during mid-swing (*p* < 0.05); reciprocal inhibitory/H-reflex measures changed after BoNT-A.
Bhakta et al. (2008) [24]	RCT	*n* = 40	Median 2.7 years (chronic)	Dysport 1000 MU divided over elbow/wrist/finger flexors	Associated-reaction force; sEMG-related motor output	Spinal	MAS; ADL interference	Peak associated-reaction force ↓ vs. placebo at week 2 (*p* = 0.005) and week 6 (*p* < 0.01); effect attenuated by week 12.
Boudarham et al. (2013) [25]	Pre–post	*n* = 14	Chronic hemiparetic stroke	RF (164 ± 50U)	EMG	Peripheral	MAS; gait	RF EMG ↓ (*p* = 0.013–0.004); MAS ↓ (*p* = 0.012); gait velocity/stride/cadence ↑ (*p* < 0.017).
Campanella et al. (2022) [26]	Pre–post	*n* = 14	Chronic post-stroke hypertonia	BB, brachialis, GC, soleus	sEMG; SWE; MHS	Peripheral	MAS	MAS ↓ (*p* = 0.0018–0.018); sEMG ↓ (*p* < 0.005); SWE ↓ (*p* < 0.003); MHS NS.
Chalard et al. (2021) [27]	Prospective observational pre–post	*n* = 12	≥6 months post-stroke (chronic)	AboBoNT-A (Dysport) to individualized elbow flexors + usual PT/self-rehabilitation	EEG movement-related beta desynchronization; elbow-flexor EMG co-contraction	Spinal	Tardieu/catch angle; FMA-UE; WMFT; AROM	Movement-related beta desynchronization changed significantly at 4w and 16w vs. baseline; elbow-flexor overactivity/co-contraction and active elbow performance changed over follow-up.
Chandra et al. (2020) [28]	Longitudinal pre–post	*n* = 8	4–12 years post-stroke (chronic)	Routine-care BoNT-A to biceps brachii; individualized dose	Voluntary RMS-sEMG; sEMG–force relation; MU amplitude distribution	Peripheral	MAS; FMA; MVC force	Force and sEMG ↓ maximally ~2–4w after injection, followed by partial recovery; several participants remained below baseline at 12w.
Chandra et al. (2022) [29]	Pre–post	*n* = 7	4–12 years post-stroke (chronic)	BoNT-A to medial/lateral biceps; individualized dose	HD-sEMG MUAP peak-to-peak amplitude; motor-unit territory area	Spinal	MAS; FMA; MVC force	Median MUAP amplitude ↑ 47 ± 9% in 5/7 and MUTA ↓ ~20 ± 2% at 2–4w; 2 participants showed MUAP amplitude reduction.
Chen et al. (2005) [30]	Pre–post	*n* = 10	Chronic post-stroke spasticity	BB/TB (57.5U)	Reflex EMG threshold	Spinal	MAS	MAS ↓ (*p* < 0.05); reflex EMG threshold maintained or ↑ in 7/10, consistent with reduced stretch-reflex excitability.
Chen et al. (2022) [31]	Longitudinal observation	*n* = 12	Chronic stroke (≥6 months)	100U BoNT-A to spastic biceps brachii	CMAP amplitude; reflex torque; voluntary force variability	Spinal	MAS; MVC force; non-reflex torque	At 3w, spasticity, strength, reflex torque and CMAP ↓ (all *p* < 0.05); values returned toward baseline by 3 months; force variability and non-reflex torque unchanged.
Cousins et al. (2010) [32]	RCT	*n* = 30 rand.; *n* = 23 ana.	Mean 23 ± 9 days; enrolled within 3 weeks of first stroke (early/subacute)	Half-dose BoNT-A, quarter-dose BoNT-A, or saline; elbow/wrist flexor muscles	sEMG during externally imposed slow passive stretch	Peripheral	ARAT; early arm recovery measures	At week 4, passive-stretch sEMG remained stable/decreased in BoNT-A arms while increasing in placebo; quantitatively usable mean ± SD data available for elbow and wrist.
Delcamp et al. (2022) [33]	Prospective cohort	*n* = 20	Chronic stroke (≥6 months)	BB, BR, BRD (150U) + rehabilitation	EEG–EMG corticomuscular coherence; co-contraction	Spinal: Supraspinal	AROM	AROM ↑ during treatment course; co-contraction and corticomuscular coupling decreased during BoNT-A efficacy period.
Fawzi et al. (2023) [34]	Pre–post	*n* = 50	Established/chronic PSS; exact time NR here	GC, soleus, BB, FCR (100U)	H-reflex	Spinal	MAS; MRC	MAS ↓ (*p* < 0.001); MRC ↑ (*p* < 0.001); H-reflex amplitude ↓ (*p* ≤ 0.002).
Fujita et al. (2019) [35]	Controlled	*n* = 34	BoNT-A: 75.2 ± 51.2 m; BoNT-A+PT: 39.8 ± 37.7 m (chronic)	GC, soleus, TP, FDL, FHL (≤300U)	Gait EMG; co-activation	Peripheral; Spinal	MAS; gait; ROM; clonus	Soleus EMG ↓ after BoNT-A; broader gait-muscle reorganization with BoNT-A+PT; gait velocity/cadence/stride ↑ with PT; MAS/clonus ↓ and ROM ↑.
Gandolfi et al. (2019) [36]	RCT	*n* = 32	Chronic stroke	BB, PM, TB, wrist flexors	sEMG	Peripheral	MAS; FMA; strength	MAS ↓ in both groups (*p* < 0.01); FMA ↑ in both groups (*p* < 0.001); biceps recruitment increased qualitatively.
Girlanda et al. (1997) [37]	Pre–post	*n* = 20	Chronic post-stroke upper-limb spasticity	FCR, FCU, BR, FDP, FDS, FPL	EMG; H/M ratio; reciprocal inhibition	Peripheral; Spinal	MAS	MAS ↓ (*p* < 0.01); Hmax ↓ (*p* < 0.01); Mmax ↓ (*p* < 0.05); normalized spinal effects limited/variable.
Hesse et al. (1996) [38]	Pre–post	*n* = 12	Chronic hemiparetic stroke	Soleus, TP, GC (400U)	EMG	Peripheral	MAS; gait; kinematics	MAS ↓ 1–2 grades; premature soleus EMG ↓ ~35%; gait velocity ↑ ~33%; dorsiflexion ROM ↑.
Hwang et al. (2024) [39]	RCT	*n* = 43	Chronic PSS	BB, BR, FCU, FCR, FDS, FDP (≤300U)	EMG (RMS)	Peripheral	MAS; VAS; K-MBI; EQ-5D; FMA-UE	MAS ↓; VAS ↓ (*p* < 0.001); K-MBI and EQ-5D ↑; EMG showed significant group × time interaction (*p* = 0.032).
Im et al. (2014) [40]	Randomized controlled	*n* = 40	Chronic stroke	GC (200U)	EMG (RMS)	Peripheral	MAS; MTS; clonus; gait	Injected-muscle RMS ↓; MAS markedly ↓; gait speed improved in one injection-site group.
Kerzoncuf et al. (2015) [41]	Longitudinal pre–post	*n* = 8	60 ± 45 months; range 29–168 months (chronic)	Botox to triceps surae; total ~250–300U; electrical-stimulation guidance	Soleus H-reflex post-activation depression	Spinal	MAS; clonus; ankle PROM; FIM; FAC; residual motor control	MAS ↓ 1 point at 3w; post-activation depression restored in participants with residual motor control but further reduced in those without; PROM improved in 6/8.
Kirazli et al. (1998) [42]	Controlled	*n* = 20	Chronic post-stroke spastic foot	Soleus, TP, MG/LG (400U)	EMG (clonus)	Peripheral	MAS; ROM; ambulation	MAS ↓ (*p* < 0.05); clonus ↓ (*p* < 0.05); ROM ↑; BoNT-A compared favorably with phenol for several outcomes.
Lee et al. (2008) [43]	Before–after longitudinal	*n* = 8	Chronic stroke; exact duration NR here	Botox to upper limb including biceps (50–100U); other flexors individualized	Reflex EMG threshold during passive stretch; viscosity index	Peripheral	MAS; biomechanical resistance	MAS and viscosity index ↓; reflex EMG threshold ↑ significantly after injection, with peak effects generally at 2–6w and variable relapse by 9w.
Liu et al. (2023) [44]	Pre–post	*n* = 8	73.1 ± 42.2 months; all ≥6 months (chronic)	100U to spastic biceps brachii	CMAP; HDWA-MUNE; SMUP amplitude	Spinal	MAS; voluntary contraction measures	CMAP ↓ from 8.55 ± 2.34 to 6.57 ± 1.92 mV (*p* < 0.02); MUNE changes heterogeneous/non-significant overall; motor-unit size/distribution differed across sides and visits.
Marchand-Pauvert et al. (2013) [45]	Pre–post	*n* = 14	Chronic stroke	Soleus, TP, GC	H/M reflex; Mmax; recurrent inhibition	Spinal	MAS	Mmax ↓ (*p* < 0.05); H-reflex amplitude largely NS; MAS ↓; recurrent inhibition was depressed after BoNT-A.
Marvulli et al. (2016) [46]	Controlled	*n* = 36	Chronic post-stroke upper-limb spasticity	FDS (~118U)	CMAP	Spinal	MAS; ROM; ARAT	MAS ↓ (*p* < 0.001); ROM ↑ (*p* < 0.001); CMAP ↓ (*p* < 0.001); ARAT ↑ (*p* < 0.001).
Miscio et al. (2004) [47]	Pre–post	*n* = 18	Chronic post-stroke spasticity	FCR, FCU, FDP, FDS	EMG; stiffness	Peripheral	MAS; BI; VAS	MAS ↓ (*p* < 0.05); stiffness ↓ (*p* < 0.001); ROM ↑ (*p* < 0.05); BI improved in 4 patients; pain improved in 3.
Nguyen et al. (2024) [48]	Pre–post	*n* = 10	Chronic post-stroke spasticity	BB	HD-sEMG during passive stretch	Peripheral	MAS	MAS ↓ (*p* = 0.0238); passive-stretch HD-sEMG biomarkers changed, while EMG slope was NS.
Pandyan et al. (2002) [49]	Pre–post	*n* = 14	Established unilateral post-stroke spasticity	BB 70U; BR 56.5U; FDL 83.3U	EMG	Peripheral	MAS; strength; ARAT	EMG ↓ (*p* < 0.05); MAS ↓ (*p* < 0.05); strength ↑ (*p* < 0.05); ARAT ↑ (*p* < 0.05).
Panizza et al. (2000) [50]	Pre–post	*n* = 15	Mean 21 months; all ≥6 months (chronic)	Botox total 80–200 IU; individualized upper-limb muscles	FCR Hmax/Mmax; H-reflex presynaptic inhibition during vibration	Spinal	Ashworth; active ROM; task score	Ashworth ↓ 3.8 ± 0.9→2.5 ± 1.0 (*p* < 0.0001); task score ↑ (*p* < 0.0014); Hmax/Mmax and presynaptic inhibition showed no significant change.
Stampacchia et al. (2004) [51]	Pre–post	*n* = 20	Chronic PSS	FDS, FDP, FCR, FCU, BB/BRD (100U)	Stretch-reflex threshold	Peripheral	MAS; ROM	Stretch-reflex threshold ↑ (*p* < 0.05); MAS ↓ ≥1 point in 16/20; ROM changes mixed.
Tang et al. (2012) [52]	Controlled	*n* = 25	Chronic post-stroke lower-limb spasticity	GC, soleus, TP (400U)	PEMG/selective motor control	Peripheral	MAS; FMA	MAS ↓ (*p* < 0.01); EMG-derived selective motor control ↑ (*p* < 0.01); FMA ↑ at 12w (*p* < 0.05).
Trompetto et al. (2008) [53]	Longitudinal pre–post	*n* = 8	Chronic post-stroke upper-limb spasticity	First BoNT-A injection; wrist/finger flexors 25–50U per muscle	Tonic vibration reflex; Mmax	Peripheral	Ashworth; MRC	Tonic vibration reflex relative to Mmax decreased after BoNT-A, supporting an intrafusal/spindle effect; clinical tone also decreased.
Veverka et al. (2016) [54]	Longitudinal fMRI pre–post	*n* = 7	7–28 months; median 10 months (chronic)	Botox 50U each to FCU, FCR, FDS, FDP + standardized physiotherapy	Task fMRI BOLD during passive wrist movement	Supraspinal	MAS; mMRC; NIHSS; BI; mRS	MAS ↓ transiently at 4w; additional bilateral cerebellar/contralesional activation emerged at 4w, with significant session contrasts in cerebellar, occipital and sensorimotor regions.
Veverka et al. (2021) [55]	Observational longitudinal	*n* = 31	3–139 months; median 10 months (chronic)	BoNT-A to affected upper limb + physiotherapy	Median-nerve cortical SEPs: P22/N30 and N20/P23	Supraspinal	MAS	Postcentral SEP amplitudes lower over affected cortex at baseline; cortical SEP components showed no significant BoNT-related longitudinal change despite clinical MAS improvement.
Veverka et al. (2023) [56]	Observational	*n* = 22 (14 vs. 8)	Chronic; ≥3 months post-stroke	Upper-limb BoNT-A	Resting-state fMRI connectivity; SEP	Supraspinal	MAS	MAS ↓ at 4w; hIP3–contralesional superior parietal connectivity increased during peak BoNT effect; connectivity related to MAS at W4.
Vinehout et al. (2021) [57]	Longitudinal fMRI	*n* = 9 stroke + 8 controls	1.1–11.9 years post-stroke (chronic)	Clinical BoNT-A for focal upper-limb spasticity; rehabilitation varied	Task fMRI BOLD activation and functional connectivity	Supraspinal	FMA; clinical spasticity measures	At 6w, activation ↑ in contralesional premotor cortex, cingulate, thalamus, superior cerebellum and ipsilesional sensory-integration cortex; connectivity related to FMA.
Wang et al. (2018) [58]	Pre–post	*n* = 21	Chronic stroke	Wrist/finger flexors; BoNT-A	NeuroFlexor; neural component; elasticity; viscosity; modeled stretch-reflex parameters	Spinal	Passive ROM	Neural component ↓ at 4w and returned toward baseline at 12w; motoneuron-pool threshold ↑ at 4w; linear stiffness and viscosity NS; nonlinear stiffness ↑ at 12w; passive ROM ↓ at 12w
Wu-Tao et al. (2015) [59]	Controlled	*n* = 23	Subacute stroke	GC 100U; soleus 50U; TP 50U	sEMG	Peripheral	MAS; FMA; gait; 6MWT	MAS ↓ (*p* < 0.05); sEMG ↓ (*p* < 0.05); gait parameters and FMA ↑ (*p* < 0.05).

Abbreviations: 6MWT, 6-Minute Walk Test; AD, anterior deltoid; ADL, activities of daily living; AboBoNT-A, abobotulinumtoxinA; ARAT, Action Research Arm Test; AROM, active range of motion; BB, biceps brachii; BI, Barthel Index; BOLD, blood-oxygen-level-dependent; BoNT-A, botulinum toxin type A; BRD, brachioradialis; CMAP, compound muscle action potential; EEG, electroencephalography; EMG, electromyography; ECR, extensor carpi radialis; EQ-5D, EuroQol 5-Dimension; FAC, Functional Ambulation Category; FCR, flexor carpi radialis; FCU, flexor carpi ulnaris; FDL, flexor digitorum longus; FDP, flexor digitorum profundus; FDS, flexor digitorum superficialis; FHL, flexor hallucis longus; FIM, Functional Independence Measure; FMA, Fugl–Meyer Assessment; FMA-UE, Fugl–Meyer Assessment–Upper Extremity; fMRI, functional magnetic resonance imaging; FPL, flexor pollicis longus; GC, gastrocnemius; GPS, Global Pain Scale; H-reflex, Hoffmann reflex; HD-sEMG, high-density surface electromyography; HDWA-MUNE, high-density weighted-average motor unit number estimation; K-MBI, Korean Modified Barthel Index; LG, lateral gastrocnemius; MAS, Modified Ashworth Scale; MG, medial gastrocnemius; MHS, muscle hardness score; Mmax, maximal M-wave amplitude; MRC, Medical Research Council scale; mMRC, modified Medical Research Council scale; MTS, Modified Tardieu Scale; MUAP, motor unit action potential; MUNE, motor unit number estimation; MUTA, motor unit territory area; MVC, maximal voluntary contraction; NIHSS, National Institutes of Health Stroke Scale; NS, not significant; NWR, nociceptive withdrawal reflex; PD, posterior deltoid; PEMG, poly-electromyography; PM, pectoralis major; PROM, passive range of motion; PSS, post-stroke spasticity; PT, physiotherapy; RF, rectus femoris; RMS, root mean square; ROM, range of motion; SEP, somatosensory evoked potential; sEMG, surface electromyography; SMUP, single motor unit potential; SWE, shear-wave elastography; TB, triceps brachii; TP, tibialis posterior; U, Units; VAS, Visual Analogue Scale; WMFT, Wolf Motor Function Test; ↓, decrease in absolute score; ↑, increase in absolute score.

**Table 2:** Methodological quality assessment of uncontrolled before–after studies using the NHLBI Quality Assessment Tool for Before–After (Pre–Post) Studies With No Control Group. Y, yes; N, no; NR, CD, not reported/cannot determine; –, not applicable. Q1–Q12 correspond to the 12 methodological criteria of the NHLBI tool. Overall study quality was judged qualitatively as good, fair, or poor and was not calculated as a numerical sum of individual criteria.

Author	Year	Q1	Q2	Q3	Q4	Q5	Q6	Q7	Q8	Q9	Q10	Q11	Q12	Overall
Albani et al. [21]	2010	Y	Y	Y	NR	N	Y	Y	NR	Y	Y	N	NA	Fair
Alvisi et al. [22]	2018	Y	Y	Y	NR	N	Y	Y	NR	Y	Y	N	NA	Fair
Aymard et al. [23]	2013	Y	Y	Y	NR	N	Y	Y	NR	Y	Y	N	NA	Fair
Boudarham et al. [35]	2013	Y	Y	Y	NR	N	Y	Y	NR	Y	Y	N	NA	Fair
Campanella et al. [26]	2022	Y	Y	Y	NR	N	Y	Y	NR	Y	Y	N	NA	Fair
Chen et al. [30]	2005	Y	Y	Y	NR	N	Y	Y	NR	Y	Y	N	NA	Fair
Fawzi et al. [34]	2023	Y	Y	Y	NR	N	Y	Y	NR	Y	Y	N	NA	Fair
Girlanda et al. [37]	1997	Y	Y	Y	NR	N	Y	Y	NR	Y	Y	N	NA	Fair
Hesse et al. [38]	1996	Y	Y	Y	NR	N	Y	Y	NR	Y	Y	N	NA	Fair
Marchand-Pauvert et al. [45]	2013	Y	Y	Y	NR	N	Y	Y	NR	Y	Y	N	NA	Fair
Miscio et al. [47]	2004	Y	Y	Y	Y	N	Y	Y	NR	Y	Y	N	NA	Fair
Nguyen et al. [48]	2024	Y	Y	CD	NR	N	Y	Y	NR	Y	Y	N	NA	Fair
Pandyan et al. [49]	2002	Y	Y	Y	N	N	Y	Y	NR	Y	Y	N	NA	Fair
Stampacchia et al. [51]	2004	Y	Y	Y	NR	N	Y	Y	NR	Y	Y	N	NA	Fair
Chalard et al. [27]	2021	Y	Y	Y	NR	N	Y	Y	NR	Y	Y	N	NA	Fair
Chandra et al. [28]	2020	Y	Y	CD	NR	N	Y	Y	NR	CD	Y	N	NA	Fair
Chandra et al. [29]	2022	Y	Y	CD	NR	N	Y	Y	NR	Y	Y	N	NA	Fair
Chen et al. [31]	2022	Y	Y	Y	NR	N	Y	Y	NR	Y	Y	N	NA	Fair
Kerzoncuf et al. [41]	2015	Y	Y	CD	NR	N	Y	Y	NR	Y	Y	N	NA	Fair
Lee et al. [43]	2008	Y	Y	CD	NR	N	Y	Y	NR	Y	Y	N	NA	Fair
Liu et al. [44]	2023	Y	Y	CD	NR	N	Y	Y	NR	Y	Y	N	NA	Fair
Panizza et al. [50]	2000	Y	Y	CD	NR	N	CD	Y	NR	CD	Y	N	NA	Poor
Trompetto et al. [53]	2008	Y	Y	CD	NR	N	Y	Y	NR	Y	Y	N	NA	Fair
Veverka et al. [54]	2016	Y	Y	CD	NR	N	Y	Y	NR	Y	Y	N	NA	Fair
Veverka et al. [55]	2021	Y	Y	Y	NR	N	Y	Y	NR	Y	Y	N	NA	Fair
Wang et al. [58]	2018	Y	Y	Y	NR	N	Y	Y	NR	Y	Y	N	NA	Fair

**Table 3:** GRADE certainty of evidence reflects how confident we are that the results are close to the true effect, rated as high, moderate, low, or very low based on domains such as: Risk of Bias, Inconsistency, Indirectness, Imprecision, Publication Bias and Overall Certainty. Publication bias was not formally assessed because fewer than 10 studies contributed to each construct; therefore, publication bias could not be excluded, but no additional downgrade was applied.

Construct	Initial Certainty	Risk of Bias	Inconsistency	Indirectness	Imprecision	Publication Bias	Final Certainty
Passive stretch-evoked responses	Low	Serious	Serious	Not serious	Serious	Not assessable	Very low
Voluntary agonist activity	Low	Serious	Very serious	Not serious	Serious	Not assessable	Very low
CMAP amplitude	Low	Serious	Not serious	Not serious	Serious	Not assessable	Very low
Reciprocal motor control	Low	Serious	Not serious	Not serious	Serious	Not assessable	Very low
Hmax/Mmax	Low	Serious	Very serious	Not serious	Serious	Not assessable	Very low
Reciprocal inhibition	Low	Serious	Not assessable	Not serious	Serious	Not assessable	Very low
Recurrent inhibition	Low	Serious	Not assessable	Not serious	Serious	Not assessable	Very low

**Table 4:** Sensitivity analysis of the pooled constructs.

Outcome Construct	r = 0.30, g (95% CI)	r = 0.50, g (95% CI)	r = 0.70, g (95% CI)
Passive stretch-evoked responses	0.80 (0.34–1.26)	0.80 (0.35–1.24)	0.79 (0.37–1.21)
Voluntary agonist activation	0.32 (−0.39–1.02)	0.31 (−0.38–1.00)	0.31 (−0.35–0.97)
CMAP amplitude	0.69 (0.16–1.22)	0.69 (0.23–1.15)	0.68 (0.30–1.07)
Reciprocal motor control	0.54 (0.24–0.84)	0.52 (0.23–0.81)	0.50 (0.22–0.77)
Hmax/Mmax	0.22 (−0.75–1.19)	0.22 (−0.74–1.17)	0.21 (−0.72–1.15)

**Table 5:** Final search strategy (18 August 2026). Note: The asterisk (*) denotes a truncation wildcard used to retrieve multiple word endings.

Table Heading	Search Strategy	Results
PubMed	(“Stroke”[MeSH] OR stroke*[tiab] OR poststroke[tiab] OR “post-stroke”[tiab] OR “cerebrovascular accident*”[tiab] OR hemipleg*[tiab] OR hemipar*[tiab]) AND (“Botulinum Toxins”[MeSH] OR “Botulinum Toxins, Type A”[MeSH] OR “botulinum toxin”[tiab] OR “botulinum toxin type A”[tiab] OR “botulinum toxin A”[tiab] OR “botulinum neurotoxin”[tiab] OR “botulinum neurotoxin type A”[tiab] OR BoNT[tiab] OR “BoNT-A”[tiab] OR BTX[tiab] OR “BTX-A”[tiab] OR Botox[tiab] OR Dysport[tiab] OR Xeomin[tiab] OR onabotulinumtoxinA[tiab] OR abobotulinumtoxinA[tiab] OR incobotulinumtoxinA[tiab]) AND (EMG[tiab] OR electromyograph*[tiab] OR myoelectric*[tiab] OR “muscle activ*”[tiab] OR “muscular activ*”[tiab] OR “motor activ*”[tiab] OR “involuntary activ*”[tiab] OR “voluntary activ*”[tiab] OR coactivat*[tiab] OR “co-activat*”[tiab] OR cocontract*[tiab] OR “co-contract*”[tiab] OR “associated reaction*”[tiab] OR reflex*[tiab] OR “H reflex”[tiab] OR “H-reflex”[tiab] OR “Hoffmann reflex”[tiab] OR Hmax[tiab] OR Mmax[tiab] OR “H/M ratio”[tiab] OR “Hmax/Mmax”[tiab] OR “stretch reflex”[tiab] OR “stretch reflex threshold”[tiab] OR “reflex threshold”[tiab] OR “tonic stretch reflex threshold”[tiab] OR TSRT[tiab] OR “tendon reflex”[tiab] OR “Achilles reflex”[tiab] OR clonus[tiab] OR “tonic vibration reflex”[tiab] OR TVR[tiab] OR “post-activation depression”[tiab] OR “postactivation depression”[tiab] OR “presynaptic inhibition”[tiab] OR “reciprocal inhibition”[tiab] OR “recurrent inhibition”[tiab] OR Renshaw[tiab] OR “Ia inhibition”[tiab] OR “Ib inhibition”[tiab] OR “motor unit*”[tiab] OR CMAP[tiab] OR “compound muscle action potential*”[tiab] OR “M-wave”[tiab] OR “M wave”[tiab] OR motoneuron*[tiab] OR “motor neuron*”[tiab] OR excitability[tiab] OR “neural excitability”[tiab] OR afferent*[tiab] OR propriocept*[tiab] OR sensorimotor[tiab] OR “sensory input”[tiab] OR “sensory feedback”[tiab] OR “transcranial magnetic stimulation”[tiab] OR TMS[tiab] OR MEP[tiab] OR MEPs[tiab] OR “motor evoked potential*”[tiab] OR corticospinal[tiab] OR intracortical[tiab] OR “cortical excitability”[tiab] OR fMRI[tiab] OR “functional MRI”[tiab] OR “functional magnetic resonance imaging”[tiab] OR neuroimag*[tiab] OR “functional connectivity”[tiab] OR “brain activation”[tiab] OR “cortical activation”[tiab] OR plasticity[tiab] OR neuroplastic*[tiab] OR corticomuscular[tiab] OR “cortico-muscular”[tiab] OR coherence[tiab] OR neurophysiolog*[tiab] OR electrophysiolog*[tiab])	365
Web of Science	(stroke OR poststroke OR “post-stroke” OR “cerebrovascular accident” OR hemiplegia OR hemiparesis) AND (“botulinum toxin” OR “botulinum toxin type A” OR “botulinum toxin A” OR “botulinum neurotoxin” OR BoNT OR BTX OR Botox OR Dysport OR Xeomin) AND (EMG OR electromyography OR electromyographic OR “muscle activation” OR “muscle activity” OR “associated reactions” OR reflex OR “H reflex” OR “Hoffmann reflex” OR Hmax OR Mmax OR “H/M ratio” OR “stretch reflex” OR “stretch reflex threshold” OR “reflex threshold” OR TSRT OR clonus OR “tendon reflex” OR “reciprocal inhibition” OR “recurrent inhibition” OR Renshaw OR “motor unit” OR CMAP OR “M wave” OR excitability OR afferent OR sensorimotor OR TMS OR “transcranial magnetic stimulation” OR MEP OR “motor evoked potential” OR corticospinal OR intracortical OR fMRI OR neuroimaging OR “functional connectivity” OR “brain activation” OR “cortical activation” OR plasticity OR neuroplasticity OR corticomuscular OR coherence OR neurophysiology OR neurophysiological OR electrophysiology OR electrophysiological)	638
Scopus	(TITLE-ABS-KEY (stroke) AND TITLE-ABS-KEY (“botulinum toxin” OR “botulinum toxin type A” OR “botulinum toxin A” OR “botulinum neurotoxin” OR Botox OR Dysport OR Xeomin OR onabotulinumtoxinA OR abobotulinumtoxinA OR incobotulinumtoxinA) AND TITLE-ABS-KEY (EMG OR electromyography OR electromyographic OR “muscle activation” OR “muscle activity” OR “motor activation” OR “motor activity” OR “voluntary activation” OR “involuntary activation” OR coactivation OR cocontraction OR “associated reaction” OR “associated reactions” OR reflex OR “H reflex” OR “H-reflex” OR “Hoffmann reflex” OR Hmax OR Mmax OR “H/M ratio” OR “Hmax/Mmax” OR “stretch reflex” OR “stretch reflex threshold” OR “reflex threshold” OR “tonic stretch reflex threshold” OR TSRT OR “tendon reflex” OR “Achilles reflex” OR clonus OR “tonic vibration reflex” OR “post-activation depression” OR “presynaptic inhibition” OR “reciprocal inhibition” OR “recurrent inhibition” OR Renshaw OR “motor unit” OR “motor units” OR CMAP OR “compound muscle action potential” OR “M wave” OR motoneuron OR “motor neuron” OR excitability OR “neural excitability” OR afferent OR afferents OR proprioception OR sensorimotor OR “sensory input” OR “sensory feedback” OR TMS OR “transcranial magnetic stimulation” OR MEP OR “motor evoked potential” OR corticospinal OR intracortical OR “cortical excitability” OR fMRI OR “functional MRI” OR “functional magnetic resonance imaging” OR neuroimaging OR “functional connectivity” OR “brain activation” OR “cortical activation” OR plasticity OR neuroplasticity OR corticomuscular OR coherence OR neurophysiology OR neurophysiological OR electrophysiology OR electrophysiological))	655
Embase	(‘stroke’/exp OR stroke*:ti,ab,kw OR poststroke:ti,ab,kw OR ‘post-stroke’:ti,ab,kw OR ‘cerebrovascular accident’:ti,ab,kw OR hemipleg*:ti,ab,kw OR hemipar*:ti,ab,kw) AND (‘botulinum toxin’/exp OR ‘botulinum toxin type a’/exp OR ‘botulinum toxin’:ti,ab,kw OR ‘botulinum toxin type a’:ti,ab,kw OR ‘botulinum toxin a’:ti,ab,kw OR ‘botulinum neurotoxin’:ti,ab,kw OR botox:ti,ab,kw OR dysport:ti,ab,kw OR xeomin:ti,ab,kw OR onabotulinumtoxina:ti,ab,kw OR abobotulinumtoxina:ti,ab,kw OR incobotulinumtoxina:ti,ab,kw) AND (emg:ti,ab,kw OR electromyograph*:ti,ab,kw OR ‘surface emg’:ti,ab,kwOR semg:ti,ab,kw OR ‘muscle activation’:ti,ab,kw OR ‘muscle activity’:ti,ab,kw OR ‘associated reaction’:ti,ab,kw OR ‘associated reactions’:ti,ab,kw OR ‘h reflex’:ti,ab,kw OR ‘h-reflex’:ti,ab,kw OR ‘hoffmann reflex’:ti,ab,kw OR ‘stretch reflex’:ti,ab,kw OR ‘stretch reflex threshold’:ti,ab,kw OR ‘reflex threshold’:ti,ab,kw OR ‘reciprocal inhibition’:ti,ab,kw OR ‘recurrent inhibition’:ti,ab,kw OR ‘presynaptic inhibition’:ti,ab,kw OR hmax:ti,ab,kw OR mmax:ti,ab,kw OR ‘h/m ratio’:ti,ab,kw OR ‘motor unit’:ti,ab,kw OR ‘compound muscle action potential’:ti,ab,kw OR ‘m wave’:ti,ab,kw OR ‘transcranial magnetic stimulation’:ti,ab,kw OR tms:ti,ab,kw OR mep:ti,ab,kw OR ‘motor evoked potential’:ti,ab,kw OR corticospinal:ti,ab,kw OR intracortical:ti,ab,kw OR fmri:ti,ab,kw OR neuroimag*:ti,ab,kw OR ‘functional connectivity’:ti,ab,kw OR ‘brain activation’:ti,ab,kw OR sensorimotor:ti,ab,kwOR afferent*:ti,ab,kw OR neuroplastic*:ti,ab,kw OR neurophysiolog*:ti,ab,kw OR electrophysiolog*:ti,ab,kw)	643

**Table 6:** Risk-of-bias assessment for randomized trials using the RoB 2 tool. Note. Domains include Bias arising from the randomization process (D1), Bias due to deviations from intended interventions (D2), Bias due to missing outcome data (D3), Bias in measurement of the outcome (D4) and Bias in selection of the reported result (D5). Results were identified as High Risk (○); Some Concerns (◐) and Low Risk (●) [25,32,36,39,40,42,59].

Author	Year	D1	D2	D3	D4	D5	Overall Risk of Bias
Bhakta et al. [24]	2008	●	◐	●	◐	●	◐
Cousins et al. [32]	2010	●	◐	○	●	◐	○
Gandolfi et al. [36]	2019	●	●	●	●	◐	◐
Hwang et al. [39]	2024	◐	◐	●	◐	●	◐
Im et al. [40]	2014	●	●	●	◐	●	◐
Kirazli et al. [42]	1998	●	◐	●	●	●	◐
Wu Tao et al. [59]	2015	●	●	●	●	●	●

**Table 7:** Risk of Bias assessment using the Newcastle–Ottawa Scale for non-randomized studies. Stars indicate the quality of each domain: Selection (max 4), Comparability (max 2), and Exposure (max 3). Note: **★** indicates an awarded point and **☆** indicates a criterion for which no point was awarded. Selection, Comparability, and Exposure/Outcome domains have maximum scores of 4, 2, and 3 points, respectively. [33,35,46,52,56,57].

Risk of Bias Assessment Tool: Newcastle Ottawa Scale					
Author	Year	Selection	Comparability	Exposure/Outcome	Total
Delcamp et al. [33]	2022	**★** **★★★**	**★** **☆**	**★★★**	8/9
Fujita et al. [35]	2019	**★** **★★★**	**★** **☆**	**★★☆**	7/9
Marvulli et al. [46]	2016	**★** **★★★**	**★** **★**	**★★★**	9/9
Tang et al. [52]	2012	**★** **★★★**	**★** **★**	**★★★**	9/9
Veverka et al. [56]	2023	**★** **★★★**	**★** **★**	**★★★**	9/9
Vinehout et al. [57]	2021	**★** **★★★**	**★** **★**	**★★★**	9/9

## Data Availability

No new data were created or analyzed in this study. Data sharing is not applicable to this article.
